# The Association Between Heart Rate Variability and Neurocognitive and Socio-Emotional Development in Nepalese Infants

**DOI:** 10.3389/fnins.2019.00411

**Published:** 2019-04-26

**Authors:** Torvald F. Ask, Suman Ranjitkar, Manjeswori Ulak, Ram K. Chandyo, Mari Hysing, Tor A. Strand, Ingrid Kvestad, Laxman Shrestha, Marita Andreassen, Ricardo G. Lugo, Jaya S. Shilpakar, Merina Shrestha, Stefan Sütterlin

**Affiliations:** ^1^Kavli Institute for Systems Neuroscience and Centre for Neural Computation, Norwegian University of Science and Technology, Trondheim, Norway; ^2^Department of Research, Innlandet Hospital Trust, Lillehammer, Norway; ^3^Child Health Research Project, Department of Pediatrics, Tribhuvan University Teaching Hospital, Kathmandu, Nepal; ^4^Department of Community Medicine, Kathmandu Medical College, Kathmandu, Nepal; ^5^Department of Psychosocial Science, Faculty of Psychology, University of Bergen, Bergen, Norway; ^6^Centre for International Health, University of Bergen, Bergen, Norway; ^7^Regional Center for Child and Youth Mental Health, NORCE Norwegian Research Center, Bergen, Norway; ^8^RG-CHaP, Department of Psychology, Inland Norway University of Applied Sciences, Lillehammer, Norway; ^9^Faculty of Health and Welfare Sciences, Østfold University College, Halden, Norway; ^10^Division of Clinical Neuroscience, Oslo University Hospital, Oslo, Norway

**Keywords:** heart rate variability, neurodevelopment, socio-emotional development, neurocognitive development, Bayley scales of infant and toddler development, vagal tone

## Abstract

**Background:**

Many young children in developing countries do not reach their developmental potential. Traditional methods for assessing developmental outcome are time consuming, thus, physiological measures that can contribute to the prediction of developmental outcomes in high risk groups have been suggested. Vagally mediated heart rate variability (vmHRV) is considered a neurophysiological or peripheral proxy for prefrontal and executive functioning and might serve as a supplement for traditional measurements of developmental status and as a potential useful risk indicator.

**Aim:**

In the present study, we wanted to describe the vmHRV in Nepalese infants and relate it to the Bayley Scales of infant and toddler development, 3. edition (Bayley-III) subscales.

**Methods:**

600 Nepalese infants were included in the study. At 6–11 and 17–24 months, we measured neurodevelopmental and socio-emotional outcomes by the Bayley-III. Inter-beat intervals were recorded at two measurement points when the children were 17–24 months.

**Results:**

There was a high intraclass correlation between HRV indices generated from the two measurement points. No significant associations between vmHRV and Bayley-III sub scales were found at any time.

**Conclusion:**

This study is the first to describe vmHRV in healthy infants and the relationship between Bayley-III scores. Our results suggest that vmHRV is not associated with measures of general development in infancy.

## Introduction

Reaching optimal neurocognitive and socio-emotional potential is a challenge for many children, and efforts to understand the process of poor developmental outcomes are necessary (McCoy et al., 2016). Available tools for measuring neurodevelopment in infants are time-consuming and physiological measures that can contribute to the prediction of developmental outcomes in high risk groups have been suggested. Vagal tone is associated with emotional, cognitive/academic, and social adaptability in children (Graziano and Derefinko, 2013). It has been suggested that vagal tone is influenced by the prefrontal cortical structures (Thayer et al., 2012; Jennings et al., 2016; Nikolin et al., 2017) that provide the neurocognitive basis for executive functions and include basic cognitive processes such as the preparation and planning for action, decision-making, cognitive-emotional regulation, and components crucial for key social behavior (Anderson, 2002). Prefrontal influences on vagal tone are proposed to be achieved by modulation of vagal activation via efferent connectivity with pre-autonomic structures (Appelhans and Luecken, 2006; Thayer and Lane, 2009) and prefrontal inhibitory control over arousal-eliciting activity in the amygdala (Golkar et al., 2012; Thayer et al., 2012). The prefrontal influences on vagal tone is suggested to be reflected in resting vagally mediated (vm) heart rate variability (HRV; vmHRV) which refers to the beat-to-beat variation in heart rate that reflects vagal activation (Task Force of the European Society of Cardiology and the North American Society of Pacing and Electrophysiology, 1996). Higher beat-to-beat variability in heart rate (high vmHRV) indicates higher prefrontally modulated vagal activation, while lower beat-to-beat variability in heart rate (low vmHRV) indicates lower prefrontally modulated vagal activation (Berntson et al., 1997; Thayer et al., 2012). Respiratory sinus arrhythmia (RSA), which refers to how the cyclical patterns of breathing modulates variation in heart rate, serves as another index of tonic vagal tone that is closely linked to trait vmHRV. Resting vmHRV is positively associated with performance on tasks requiring executive control (Thayer and Lane, 2009) and is considered reflecting self-regulatory strength in infants and children (Bornstein and Suess, 2000; Griffiths et al., 2017; but see also Zahn et al., 2016).

High vmHRV is associated with fewer self-reported emotional problems in youth (Vasilev et al., 2009) while low vmHRV is associated with socio-emotional and behavioral problems in young children and adolescents (Calkins et al., 2006; Scott and Weems, 2014; Schoorl et al., 2015). Furthermore, low vmHRV is associated with social disengagement among children in response to negative affect (Shahrestani et al., 2014). These previous findings indicate that vmHRV could be a possible predictor of socio-emotional developmental outcomes. However, as a substantial proportion of the concurrent literature on vmHRV in children employs either an emotion regulatory approach or an approach that assesses stress and health, a longitudinal approach assessing socio-emotional outcomes in young children is less explored.

In fetuses with congenital heart disease, low total HRV (indicated by interquartile range and standard deviation of RR intervals) at 34–38 weeks predicted lower cognitive and motor performance at 18 months (Siddiqui et al., 2017). In preterm and full-term infants, and in healthy infants and infants with perinatal asphyxia, high vmHRV measured at 40 weeks of gestation predicted better outcome on the mental developmental index of the Bayley Scales of Infant and Toddler Development 2nd ed. at 8 and 12 months, while neonates with low vmHRV had varied outcomes (Fox and Porges, 1985). In infants aged 6 months, RSA was associated development at that age as well as developmental outcome at 12 months (Richards and Cameron, 1989). RSA predicts better cognitive, motor, and behavioral regulatory outcomes in preterm infants with very low birth weight (Doussard-Roosevelt et al., 1997). In healthy infants, fetal total HRV (standard deviation of heart rate per minute epochs) was associated with mental and psychomotor ability at 2 years, and language ability at 2.5 years (DiPietro et al., 2007). Hence, we argue that vmHRV could also be a predictor of cognitive developmental outcomes.

In summary, vmHRV is thought to reflect the integrity of prefrontal cortical structures that is also responsible for executive cognitive functions and socio-emotional functioning. A number of studies have found associations between vmHRV and developmental outcomes in various infant populations, thus, we argue that vmHRV could be associated with cognitive and socio-emotional developmental outcomes.

Infections, poor nutrition and poor access to health care services are known risk factors for children in Nepal. Micronutrient deficiencies are common (Ulak et al., 2016; Chandyo et al., 2018). In combination with poor access to health care services to help monitor and assist in children’s development, children are at increased risk for infections (Strand et al., 2007), poor growth (Strand et al., 2015), and neurodevelopmental delay (McCoy et al., 2016).

In the present study, we want to assess vmHRV levels in a sample of Nepalese infants. We also want to examine the relationship between HRV and neurodevelopmental and socio-emotional outcomes. On accord with findings from previous studies examining HRV in relation to developmental outcome (e.g., Doussard-Roosevelt et al., 1997; Siddiqui et al., 2017), social and emotional functioning (Calkins et al., 2006; Schoorl et al., 2015), and self-regulatory functioning (Bornstein and Suess, 2000; Thayer and Lane, 2009), we hypothesize that vmHRV will be positively associated with neurocognitive and socio-emotional developmental outcomes at two different time frames of a 1 year interval.

## Materials and Methods

### Study Population

The study site is the urban and surrounding communities of Bhaktapur municipality in Nepal. The current sample is part of a randomized, double blinded, placebo controlled trial assessing the effect of vitamin B_12_ supplementation on infant growth and development (Strand et al., 2017). Thus, the present study is not a population based study, and subsequent results should be interpreted accordingly. Six hundred Nepalese children (male = 309) aged 6–11 months (*M* = 8.0, *SD* = 1.8) were enrolled in the study (Table 1). Inclusion criteria were having a length for age less than -1 *z*-score, and residing in the Bhaktapur municipality and surrounding areas, planning to stay for the next 12 months and caregivers available for informed consent. Exclusion criteria were severe systemic illness requiring hospitalization, severe malnutrition, lack of consent, taking B vitamin supplements that included vitamin B12, severe anemia (Hb < 7 g/dl), or ongoing acute infection such as fever or infection that required medical treatment. Age at end study was 17–24 months (*M* = 19.9, *SD* = 1.8). Baseline population characteristics are published elsewhere (Chandyo et al., 2018; Ranjitkar et al., 2018).

**Table 1:** Baseline characteristics of study sample (*N* = 441).

Characteristics	Number	Prevalence (%)	Mean	SD
Mean age of child (months)			8.0	1.8
Male child	231	52.4		
Birth weight, gm^1^			2787	497
Preterm birth	45	10.2		
Low birth weight (<2500 gm)	78	17.7		
Hospitalization at 1st month of age	18	4.1		
**Demographic features:**				
Mother’s age			27.6	4.6
Father’s age^2^			27.7	9.6
Literacy of mother:				
Illiterate or up to grade 5	166	37.6		
Grade 5 and above	275	62.4		
Literacy of father:				
Illiterate or up to grade 5	143	32.4		
Grade 5 and above	298	67.6		
Occupation of mother:				
No working mother/agriculture	276	62.6		
Employed	165	44.3		
Occupation of father:				
No working/agriculture	22	4.9		
Employed	419	95.1		
Ethnic group:				
Newar	316	71.7		
Lama/Tamang	66	14.9		
Brahman/Chhetri	31	7		
Other	28	7.4		
**Socio-economic status**				
Family staying in joint family	209	47.4		
Family residing in rented house	188	42.6		
Number of rooms in use by the household (≤2)	244	55.3		
Kitchen and bedroom same	212	48.1		
Family having own land	200	45.7		
Remittance from abroad	37	8.4		

*^1^among 579 infants whom birth weights were recorded. ^2^among 487 fathers who were available.*

### Research Design, Material and Procedure

#### Outcomes

##### Heart rate variability

Cardiac activity was recorded when the infants were 17–24 months. The reason why HRV was not assessed when the children were 6–11 months was that HRV could not be included due to organizational constraints in the overall research program. Cardiac activity was recorded using Alive Software (AliveTM by Somatic Vision, Inc.), which is a biofeedback system that measures heart rate through the recording of photoplethysmography (PPG; also known as blood volume pulse). Three IOM finger sensors were placed on the participant’s non-dominant hand, and PPG was recorded during a 5 to 9-min period where the participant was either awake or asleep. Very active children were assessed both while napping and while awake to assure valid recordings. Less active children were assessed only while awake. At the time of measurement, the infants were watching one of two movies; Dream House, which has very calming music, and Alien Shuttle, which is very demanding of attention. The children only watched one of the two movies. PPG was recorded twice within the same hour for every participant, with 5 min between each recording. This is in line with recommendations to use at least two measurements when using HRV as a consistent biomarker or trait, to reduce the situational impact (Bertsch et al., 2012). We were unable to obtain PPG recordings from 47 infants; 26 failed recordings were due to drop out, and the remaining 21 due to difficulty with stabilizing children for PPG assessment, postponing PPG recording but not attending later, migration, and rejection of recording activity.

Inter-beat intervals were extracted via R-peak detection and heart rate variability was analyzed using ARTiiFACT software (Kaufmann et al., 2011). Corrections of artifacts was done both through software detection and follow-up visual inspection, erroneous inter-beat intervals were replaced by means of cubic spline interpolation of neighboring intervals and deletion. Deletion was used on files that had data points with identical consecutive inter-beat intervals as that type of artifact is likely to occur where contact is lost during recording of PPG, and cubic spline interpolation does not correct those types of artifacts. Batch option for analysis of HRV was then used to generate HRV indices.

Several HRV indices were extracted, although the root Mean Square of Successive Differences (RMSSD), a time domain measure, and the High Frequency component (HFms^2^, 0.15–0.40 Hz), a frequency domain measure, were the indices of interest for analysis. Those indices primarily reflect vagally mediated parasympathetic influences on the heart (Task Force of the European Society of Cardiology and the North American Society of Pacing and Electrophysiology, 1996; Thayer et al., 2012), are highly correlated (Goedhart et al., 2007), although RMSSD might be influenced by sympathetic activation (Berntson et al., 2005).

Any recording with >30 artifacts were re-processed manually in ARTiiFACT. After re-processing, most within participant RMSSD indices became close to identical, regardless if one or both RMSSD indices were reprocessed. Deletion of inter-beat intervals reduces the duration of the total HRV recording. HRV parameters were excluded from analysis if having a recording length below 250 s before or after processing. Participants were excluded if both HRV recordings were below this cut off. 95 participants were excluded on the account of having recording lengths <250 s, reducing the remaining sample to 441 infants (age 17–24 months, *M* = 20, *SD* = 1.8; male = 231).

###### Effects of age, sex prematurity, and low birth weight on HRV

In healthy children, HRV changes a lot from early neonatal stages and throughout childhood and is thus dependent on age (Massin and von Bernuth, 1997; Silvetti et al., 2001; Bobkowski et al., 2017). HRV in young children show less test-retest reliability when the age span of the sample is wide (e.g., 2 months to 5 years, 3–5 years; Weiner and McGrath, 2016). The literature is incongruent with regards to the effect of sex on HRV in infants (e.g., Tibu et al., 2014; Bobkowski et al., 2017), although sex seems to have less impact when infants are healthy (Bobkowski et al., 2017). Moreover, there are reports that HRV is different among infants who are premature (Cabal et al., 1980; Yiallourou et al., 2013) and have low birth weight (e.g., Rakow et al., 2013). A number of the children in our sample had low birth weight (17.7%) and were preterm (10.2%). Thus, birth weight, sex, age, and whether children were preterm were included in the analysis.

##### Neurodevelopment

Neurodevelopmental outcomes were assessed using the Bayley Scales of infant and toddler development, 3rd edition (Bayley-III) when the infants were 6–11 months and again at 17–24 months (Bayley, 2006). The Bayley-III is a comprehensive assessment tool of global developmental functioning in infants and toddlers aged 1–42 months (Bayley, 2006; Weiss et al., 2010). The test takes 45 to 60 min to administer, and consists of three domains; the Cognitive, Language (receptive and expressive communication) and Motor (fine and gross motor) domain. Each test item is scored “credit” or “no credit” according to the manual, and the credited scores are summed for the total raw scores for each scale.

The Bayley raw scores are converted into standard scaled scores based on the American norms (Bayley, 2006). The standardized scaled scores have a mean of 10, a standard deviation of 3, and a range from 1 to 19. Inter-rater reliability was assessed in a previous study (Ranjitkar et al., 2018); in general, coefficients were excellent for both the standardization sample and for the quality control throughout the study, and Cronbach’s alphas for internal consistency measures ranged between 0.57 and 0.87. See Ranjitkar et al. (2018) for details on training and procedures and the cultural aptness of the Bayley-III as a tool for assessment of neurodevelopment in the current sample.

Children were tested in the presence of their mothers or another caregiver. Ahead of testing, we ensured that the children were well fed and not sick. The testing was done at the field office in a room that is well-lit, well-ventilated, and free from any distractions. The testing was conducted by an examiner that spoke the infant’s native language. The examiner started with rapport building with the child while the mother or caregiver was instructed about the assessment, followed by the test-administration according to the manual. Breaks were given during the assessment for feeding, rest, and/or nap when needed. The number of breaks were not fixed and varied according to the child’s need. Specially, cognitive, language, and fine motor subtests were carried out on the table sitting on the mother’s lap in front of the assessor, while the gross motor subtest was assessed on the floor.

Socio-emotional functioning was assessed based on caregiver’s report using the Bayley-III Social-Emotional scale at 6–11 months and at 17–24 months (Bayley-SE; Weiss et al., 2010). The scale is an adaptation of the Greenspan Social-Emotional Growth Chart: A Screening Questionnaire for Infants and Young Children (Greenspan, 2004). The questionnaire was initially adopted for the Malnutrition and Enteric diseases (Mal-ED) study (Murray-Kolb et al., 2014) in the same population of children from 6 to 24 month in which translation and back translation procedures were followed according to standard guidelines. The Bayley-SE focuses on the acquisition of broad socio-emotional milestones in infants and toddlers aged 1–42 months. The following six ratings are selected for each item; how often the caregiver observed the behavior of his/her child for scoring: 0 (Can’t tell), 1 (None of the time), 2 (Some of the time), 3 (Half of the time), 4 (Most of the time), or 5 (All of the time). The raw scores were converted into scaled score based on American norms having a mean of 10, a standard deviation 3, and a range from 1 to 19.

Index scores are commonly reported for children in our age group. In the present study, as we were specifically interested in social-emotional and neurocognitive development, Bayley-III subscales were assessed while index scores for language, motor, and cognition were not.

### Statistical Analysis

Demographic characteristics of children were summarized using means and standard deviations (SD) for continuous data, and using frequency counts and percentages for categorical data (Table 1). vmHRV indices were summarized using minimum value, maximum value, means, and SD.

**FIGURE 1:**
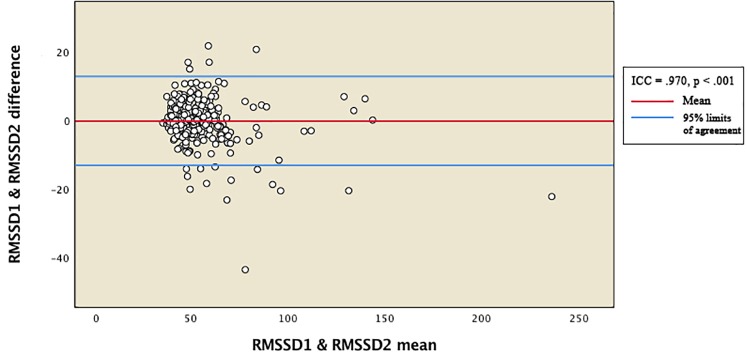
Bland-Altman plot for total distribution of RMSSD. RMSSD1 and RMSSD2 represent RMSSD indices generated from two separate within-participant recordings of HRV. ICC, intraclass correlation coefficients.

**FIGURE 2:**
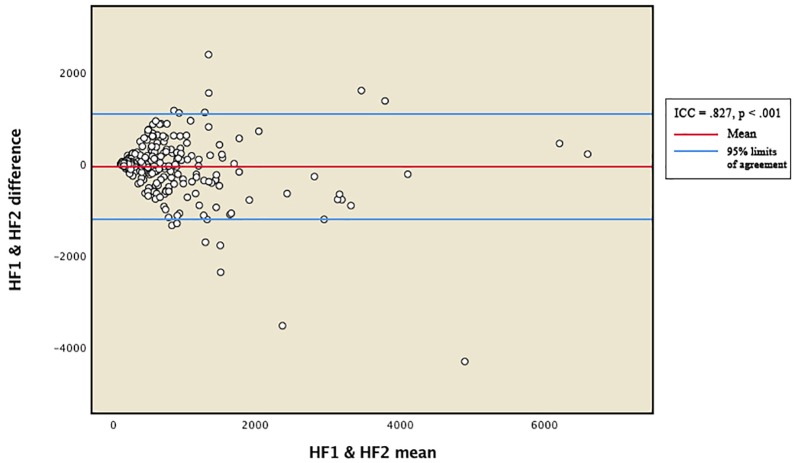
Bland-Altman plot for total distribution of HFms^2^. HF1 and HF2 represent HFms^2^ indices generated from two separate within-participant recordings of HRV. ICC, intraclass correlation coefficient.

Reliability between the vmHRV measures was expressed by the intraclass correlation coefficient (ICC). Total distribution of RMSSD and HFms^2^ scores from the two measurement points were presented in Bland-Altman plots (Figure 1, 2). The 95% limits of agreement for the total RMSSD and HFms^2^ distributions were generated by multiplying the standard deviation of the score difference with 1.96 as a parenthetical expression, then adding that expression to the mean of the difference to create the upper interval, and subtracting the expression from the mean of the difference to create the lower interval. Mean vmHRV variables were generated for RMSSD and HFms^2^. Visual and statistical inspection of HFms^2^ and RMSSD revealed that these variables were not normally distributed. Associations between vmHRV measures and Bayley-III scores were expressed using Spearman’s rho coefficients. Mean differences in vmHRV between ages at time of PPG recording (differences between children aged 17, 18, 19, 20, 21, 22, 23, and 24 months at time of recording) were analyzed using one way ANOVA. Multiple regression was conducted controlling simultaneously for age at time of PPG recording, sex, low birth weight, and whether children were preterm. Data was analyzed using SPSS version 25 (IBM Corp, 2017).

## Results

### Demographic Characteristics and Descriptive Statistics of HRV Variables

The demographic characteristics of the studied population (*N* = 441) are shown in Table 1. The sample comprised mainly of children from the Newar (71.7%) and Tamang/Lama (14.9%) ethnic groups. 45 infants (10.2%) were preterm (gestation before 37 weeks), and 78 children (17.7%) had a birth weight below 2500 g.

Table 2 provides descriptive statistics for all extracted HRV variables. Visual representation of the total distribution of RMSSD and HFms^2^ scores from both recordings are provided in Figure 1, 2. Due to high ICC (Cicchetti, 1994) between RMSSD variables (*r* = 0.970, *p* < 0.001), mean HR variables (*r* = 0.965, *p* < 0.001), pNN50 variables (*r* = 0.953, *p* < 0.001), and HFms^2^ variables (*r* = 0.872, *p* < 0.001) calculated from the first and second recording of PPG, mean variables were generated for those parameters. Due to only moderate correlation between LFms^2^ parameters (*r* = 0.594, *p* < 0.001), scores generated from those PPG recordings are listed as separate variables in Table 2. RMSSD or HFms^2^ were not significantly different between children of different ages at the time of PPG recording (17–24 months). Table 3 provides descriptive statistics for performance on the Bayley-III subscales at both times of measurement. The infants improved on all Bayley scales from measurement at age 6–11 months (time 1) to measurement at age 17–24 months (time 2), except on the cognitive scaled score, where there is a 1.2-drop in mean score from time 1 to time 2.

**Table 2:** Descriptive statistics for HRV measures (*N* = 441).

Variables	Minimum	Maximum	Mean	Std. deviation	N
RMSSD	31.4	231.9	49.8	17.7	–
Mean HR	75.9	164.8	118.7	14	–
pNN50	2.2	80.9	28	13.3	–
LFms^2^_time 1_	8.2	12248.9	663.7	1165.7	390
LFms^2^_time 2_	13.7	8073.3	619.6	851.4	355
HFms^2^	18.3	6485.4	560.1	818.9	–

*RMSSD, Root mean square of the successive differences; pNN50, percentage of successive RR intervals that differ by more than 50 ms; LF, low frequencies; HF, high frequencies. N is only indicated for LF _time 1_ and _time 2_ because when not averaged, each measurement point alone consists of a sample size below the total of N = 441.*

**Table 3:** Descriptive statistics of Bayley-III subscale scores (*N* = 441).

Variables	Minimum	Maximum	Mean	Std. deviation
Cognitive^time 1^	2	*15*	9.5	2
Receptive^time 1^	2	14	7.7	2.1
Expressive^time 1^	3	14	7.3	1.9
FMotor^time 1^	1	17	9.5	2.3
GMotor^time 1^	1	16	8.9	2.6
SocioEm^time 1^	1	18	10.7	3.2
Cognitive^time 2^	3	13	8.1	1.6
Receptive^time 2^	1	16	8.9	2.4
Expressive^time 2^	1	15	8.5	2.5
FMotor^time 2^	7	19	10.8	1.7
GMotor^time 2^	1	19	9.1	1.8
SocioEm^time 2^	2	19	10.7	3.3

*Cognitive, cognitive scale; Receptive, receptive communication scale; Expressive, expressive communication scale; FMotor, fine motor scale; GMotor, gross motor scale; SocioEm, social emotional scale; ^time 1^, 6–11 months of age; ^time 2^ = 17–24 months of age.*

### Relationship of Vagally Mediated HRV and Bayley-III Scores

HFms^2^ and RMSSD were highly correlated (*r* = 0.786, *p* < 0.001). Table 4 provides correlation between vmHRV (RMSSD and HFms^2^) indices and Bayley-III scores. There were no significant correlations between RMSSD on any of the Bayley-III subscales at baseline when the infants were 6–11 months and at end study when the infants were 17–24 months. No significant associations were found between HFms^2^ and Bayley-III subscales at the two time points. Table 5 provides results for the regression model controlling for all confounders. When controlling for age, sex, low birth weight, and preterm children, RMSSD (β = 0.123, *R*^2^ adjusted = 0.006, *p* = 0.01) and HFms^2^ (β = 0.094, *R*^2^ adjusted <0.000, *p* = 0.049) became significant predictors of socio-emotional outcome at time 2 (17–24 months).

**Table 4:** Correlations between HRV and Bayley-III scales.

		Cog^time 1^	Rec^time 1^	Exp^time 1^	FMot^time 1^	GMot^time 1^	SocEm^time 1^	Cog^time 2^	Rec^time 2^	Exp^time 2^	FMot^time 2^	GMot^time 2^	SocEm^time 2^
RMSSD	Spearman’s rho	−0.034	−0.069	0.046	−0.017	0.066	−0.067	0.003	0.001	0.004	−0.005	0.010	0.043
	Sig. (2-tailed)	0.475	0.149	0.337	0.721	0.167	0.161	0.953	0.988	0.939	0.922	0.828	0.372
HFms^2^	Spearman’s rho	−0.044	−0.093	−0.003	0.043	0.013	−0.079	−0.028	−0.041	−0.018	−0.030	0.039	0.020
	Sig. (2-tailed)	0.362	0.050	0.945	0.367	0.791	0.096	0.556	0.395	0.0704	0.533	0.416	0.681

*Cog, cognitive scale; Rec, receptive communication scale; Exp, expressive communication scale; FMot, fine motor scale; GMot, gross motor scale; SocEm, social emotional scale; ^time 1^, 6–11 months of age, ^time 2^, 17–24 months of age; RMSSD, root mean square of the successive differences; HF, high frequencies.*

**Table 5:** Regression analysis of the relationship between HRV and Bayley-III subscales (*N* = 441).

	RMSSD			HFms^2^		
Variables	*β* stand.	*R*^2^ adjust.	*Sig.*	*β* stand.	*R*^2^ adjust.	*Sig.*
Cog^time1^	−0.035	0.057	0.453	0.000	0.056	0.992
Rec^time1^	−0.061	0.211	0.149	−0.077	0.213	0.070
Exp^time1^	0.043	−0.002	0.373	0.040	−0.002	0.403
FMot^time1^	0.030	0.027	0.523	0.080	0.032	0.088
GMot^time1^	0.026	0.082	0.569	−0.020	0.082	0.657
SocEm^time1^	−0.067	0.009	0.162	−0.065	0.009	0.173
Cog^time2^	0.002	0.049	0.970	0.007	0.049	0.882
Rec^time2^	0.008	0.027	0.859	−0.026	0.028	0.575
Exp^time2^	0.049	0.044	0.298	0.018	0.042	0.695
FMot^time2^	−0.017	0.007	0.728	−0.049	0.010	0.303
GMot^time2^	0.077	−0.005	0.109	0.092	−0.003	0.056
SocEm^time2^	0.123	0.006	0.010^∗^	0.094	0.000	0.049^∗^

*All relationships controlled for birth weight in grams, sex, age, and whether children were preterm. RMSSD, root mean square of successive differences; HFms2, high frequency component; Cog, cognitive scaled score; Rec, receptive scaled score; Exp, expressive scaled score; FMot, fine motor scaled score; GMot, gross motor scaled score; SocEm, social emotional scaled score; ^time^^1^, 6–11 months of age; ^ttime 2^, 17–24 months of age. HRV was obtained at 17–24 months.*

## Discussion

The present study is to the best of our knowledge novel in terms of examining the relationship between vmHRV and the Bayley-III subscales in infants without any acute or chronic illnesses. Previous studies assessing the HRV indices we extracted in children of our age group either tend to include a wider range of ages (e.g., 0–2 years, 1–5 years; Finley and Nugent, 1995; Silvetti et al., 2001); or do not report normative values for the indices we extracted (Massin and von Bernuth, 1997; DiPietro et al., 2007). Thus, our age group (17–24 months) is arguably more discretely defined than age groups in previous studies reporting normative values for the HRV indices we have extracted. There were no mean differences in vmHRV between ages (17–24 months), and no overall association between vmHRV and neurodevelopmental outcomes and socio-emotional milestones as measured by the Bayley-III in our initial analysis. When controlling for age, sex, low birth weight, and preterm children, vmHRV became a significant predictor of socio-emotional milestones at time 2, although the effect sizes suggest that it is not very relevant for our results. It is possible that these findings contribute on a conceptual/theoretical level, for example regarding cumulative effects, but does in our opinion not have practical implications.

Efforts to describe the indices as they occur in samples of the current age group are essential in establishing a foundation for evaluating HRV as a health indicator in equivalent samples with the possible additional benefits of establishing a comparative framework for prospective measures. In the present study, we have described several commonly used indices, in addition to mean heart rate for the entire sample. In accordance with previous studies (Goedhart et al., 2007), RMSSD and HFms^2^ were highly correlated, and all HRV indices generated from the separate PPG recordings displayed excellent intraclass correlation.

We assessed whether vmHRV could be used as a neurophysiological risk indicator of developmental status. Our results indicate that there is no association between vmHRV and Bayley-III. Our findings are in contrast to previous findings indicating an association between HRV and neurodevelopmental outcome (Richards and Cameron, 1989); HRV as a predictor of cognitive and motor development in infants with congenital heart disease (Siddiqui et al., 2017); HRV as a predictor of mental developmental outcome in both healthy infants, and preterm infants and infants with perinatal asphyxia (Fox and Porges, 1985); fetal HRV as a predictor of mental and psychomotor ability at 2 years, and language ability at 2.5 years (DiPietro et al., 2007); and RSA as a predictor of better cognitive, motor, and behavioral regulatory outcomes in preterm infants with very low birth weight (Doussard-Roosevelt et al., 1997).

Several factors are possible contributors to these incongruent findings, the first being the instruments applied for assessment of developmental outcome. Only Siddiqui et al. (2017) used the Bayley-III for outcome measurements whilst earlier studies have used other tools of assessment (Fox and Porges, 1985; Richards and Cameron, 1989; Doussard-Roosevelt et al., 1997; DiPietro et al., 2007). A second reason could be timing of inter-beat interval recording; in our study we recorded inter-beat intervals at 17–24 months, which was at the end of the study, while previous studies recorded inter-beat intervals either prenatally in mothers and not the infant (Siddiqui et al., 2017) or between 14 and 40 weeks after gestation (Fox and Porges, 1985; Richards and Cameron, 1989; Doussard-Roosevelt et al., 1997), although DiPietro et al. (2007) recorded inter-beat intervals at several points of measurement; prenatal cardiac activity was recorded in mothers, while direct measures of cardiac activity in children was recorded at 2 and 2.5 years. A third possible reason could be the variation in study population, whereas previous studies have largely been comprised of infants with medical conditions (Fox and Porges, 1985; Doussard-Roosevelt et al., 1997; Siddiqui et al., 2017). HRV is related to several medical conditions and sometimes inversely related to severity (Gang and Malik, 2003) which could indicate a more prominent and perhaps confounding role in neurodevelopment under pathological circumstances that do not necessarily generalize to healthy/sub-pathological populations. In pathological samples, there is a disruptive element owed to the condition that does not occur in healthy populations. It might be the ability to handle this disruptive element in pursuit of developmental attainment that facilitates the association with vmHRV observed in previous studies. A fourth reason is that our study and previous studies differ substantially in terms of sample size. The numbers of participants in previous studies have been relatively low, our study was comprised of a total of 441 infants which would make it easier for us to detect associations.

In their meta-analysis of vmHRV in relation to self-regulation, Zahn et al. (2016) found only a weak average effect size (*r* = 0.15) and reported that there was a non-significant relationship between self-control domains, executive functions, and effect size magnitude. They also reported evidence of considerable publication bias, and that adjustment for missing studies reduced the effect size drastically resulting in a non-significant effect size close to zero. Albeit publication bias was reduced after exclusion of an outlier case, higher study quality was generally associated with lower effect size, and the authors assumed that the true effect size was below *r* = 0.15 (Zahn et al., 2016). Consequently, the possibility for publication bias in previous studies that assessed vmHRV in relation to self-regulatory domains cannot be excluded. The two measurement points of cardiac activity applied in the current study reduce the probability of state-influences on vmHRV that might have benefitted 1-measurement studies (Bertsch et al., 2012); a child at a higher developmental stage will be less nervous; a less mature child will be more nervous, thus enhancing potential situational effects imposed by mental and physical states. In a 2-measurement design the trait component is higher (Bertsch et al., 2012), thus reducing type-I-error probability. Our null-findings are in support of the findings of Zahn et al. (2016).

### Limitations

The present study has some limitations. Participant 51 through 600 (91.6%) was processed through batch option in ARTiiFACT. The drawback from this option is that one cannot select a portion of the recording that excludes researcher interference (entering the room, exiting the room, etc.) at the beginning and end of the recording. Instead the entire recording is processed. In order to counter consequences of this problem and to ensure high-quality data, recordings that had many artifacts were re-processed through both software and manually by visual inspection, and participants with recording lengths below 250 s after re-processing were excluded.

Including children of this age is a challenge in psychophysiological settings. The two films the children watched while PPG was recorded differed in emotional content; one was calming, the other was demanding of attention. This could have affected recordings. However, high intraclass correlation coefficients between same indices generated from separate recordings, and high correlations between RMSSD and HFms2 indicate that the recording conditions did not have any meaningful impact on the extracted indices. The decision was a trade-off in full consideration of the potential confounders (that were shown to be minimal). This way of showing more flexibility made it possible to include a representative group and a large sample size, without having to exclude children systematically with the price of limiting the external validity and the practical implications of the study.

Another limitation is that the current sample is a high-risk sample that is part of a clinical trial, and thus, it is not a population-based sample. Although the children improved on most of the Bayley scales from the first assessment at 6–11 months to the second assessment at 17–11 months, there was a slight drop in mean score on the cognitive scale between the two time points. Therefore, care should be taken before generalizing our results to the population.

## Conclusion

Measuring vmHRV in infants aged 17–24 months is feasible, and it is possible to secure quality vmHRV indices from 7 min inter-beat interval recordings in a sample of our magnitude (*N* = 441). This is partly reflected in the fact that the RMSSD and HFms^2^ indices in our sample were highly correlated, and the fact that we obtained excellent ICC between vmHRV indices generated from separate PPG recordings. According to our results, vmHRV is not associated with measures of general development in infancy. Controlling for sex, age, preterm children, and low birth weight does not seem to have a relevant impact on this conclusion.

## Ethics Statement

This study was carried out in accordance with the recommendations of Nepal Health Research Council (NHRC; number 233/2014) and the Regional Committee for Medical and Health Research Ethics (REC; number 2014/1528) in Norway. The protocol was approved by the Nepal Health Research Council (NHRC; number 233/2014) and the Regional Committee for Medical and Health Research Ethics (REC; number 2014/1528) in Norway. The parents/guardians of infants eligible for the trial were asked for written informed consent or for a thumbprint in the presence of an impartial witness if they were illiterate, declaring their willingness to have their infant participate in the trial.

## Author Contributions

TS, MH, IK, RC, MA, RL, and SS designed the study. RC, MU, SR, LS, JS, and MS conducted the research and were responsible for the field implementation and data collection. TS obtained funding. TA processed the HRV data and had primary responsibility for the final content. TA and TS analyzed the data and interpreted the results. All the authors read and approved the final manuscript.

## Conflict of Interest Statement

The authors declare that the research was conducted in the absence of any commercial or financial relationships that could be construed as a potential conflict of interest.
